# Effect of Multiple Reverse Transformation Treatments on Grain Refinement and Mechanical Properties of Biomedical Co–Cr–Mo–N Alloys Fabricated by Electron Beam Melting

**DOI:** 10.3390/ma16196528

**Published:** 2023-10-01

**Authors:** Hao Wang, Toshimi Miyagi, Akihiko Chiba

**Affiliations:** 1 New Industry Creation Hatchery Center (NICHe), Tohoku University, 2-1-1 Katahira, Aoba-ku, Sendai 980-8577, Japan; hao.wang.e4@tohoku.ac.jp; 2 Graduate School of Engineering, Tohoku University, 6-6, Aramaki Aza Aoba, Aoba-ku, Sendai 980-8579, Japan

**Keywords:** biomaterial, Co–Cr–Mo–N, reverse transformation treatment, mechanical property, grain refinement

## Abstract

We investigated the improvement of mechanical properties of biograde Co–28Cr–6Mo–0.11N alloy prepared by electron beam melting through grain refinement via multiple reverse transformations. While the effects of single and double reverse transformation treatments on the microstructure have been investigated in previous studies, we investigated the effects of multiple reverse transformation heat treatments. The particle size was refined to 1/4, and the yield strength, tensile silence strength, and elongation were enhanced to 655 MPa, 1234 MPa, and 45%, respectively, satisfying ASTM F75 standards. Moreover, a mixed phase of ε and γ was observed to provide higher yield strength than a single γ structure. The dominant behavior in the γ → ε phase transformation at 1073 K was obvious. Grain growth was suppressed by the grain-boundary pinning effect of the Cr_2_N phase during reverse transformation to the γ phase. Because no fracture was caused by precipitates such as σ, η, and Cr_2_N phases, the influence of the precipitates on the tensile properties was small.

## 1. Introduction

Metal alloys are often used as the base materials in biomedical devices and implants. Representative bioimplants include artificial joints for patients whose bones are damaged owing to aging or accidents. The metal materials predominantly used for artificial joints are cobalt–chromium–molybdenum alloys owing to their excellent mechanical properties, corrosion resistance, and biocompatibility [1,2,3,4]. Co–Cr–Mo alloys are known to have two structures: face-centered cubic (FCC) γ-phase at high temperatures and hexagonal close-packed (HCP) ε-phase at low temperatures. Many research results have demonstrated that adding nitrogen considerably changes the phase transformation behavior. For instance, many reported that the Co–Cr–Mo–N alloy stabilized the γ-phase even at room temperatures [5,6,7]. Kurosu et al. reported that the mechanical properties and grain refinement of the Co–27Cr–6Mo–0.16N alloy were improved because of a reverse transformation treatment that transforms the ε-phase into the γ-phase through only heat treatment [8,9]. Therefore, nitrogen addition and reverse transformation treatment are important for obtaining fine-grain materials with excellent mechanical properties of Co–Cr–Mo–N alloys.

Research on additive manufacturing of Co–Cr–Mo alloys has mainly focused on powder-bed fusion processes with laser (LBM) and electron beam melting (EBM) [10,11,12]. Kajima et al. [13] investigated the influence of anisotropy of Co–Cr–Mo samples produced by LBM varying angles between the build and longitudinal directions. Atapour et al. [14] explored the corrosion resistance, bioactivity, biocompatibility, and microstructure of LBM CoCrMo (low carbon content, heat-treated) in the XY (perpendicular) and XZ (parallel) planes of the building direction for as-built and abraded surfaces. Sun et al. [15] investigated the build direction dependence of a microstructure and its high-temperature tensile property. Nevertheless, conventionally manufactured Co–Cr–Mo–N alloys are usually strengthened through deformation mechanisms, such as work hardening (strain strengthening) using strain-induced dislocations [16,17]. However, because EBM is known as near-net-shape technology, a majority of the conventional strengthening mechanisms are not feasible. Therefore, reverse-transformed Co–Cr–Mo–N alloys fabricated using EBM are ideal for solving the difficulty of strengthening near-net-shape structures with complex geometries. Accordingly, there are reports investigated the effect of single and double reverse transformation treatments on the mechanical properties of Co–Cr–Mo–N samples produced by EBM [18].

In previous studies, only the changes in the mechanical properties of the microstructure obtained using a one-time reverse transformation treatment were investigated. Therefore, the understanding of the relationship between grain refinement and mechanical properties during multiple reverse transformation treatments is limited. The objectives of this study were to investigate the effects of the grain size, constituent phases, anisotropy, and precipitate distribution on the mechanical properties of a Co–Cr–Mo–N alloy produced by applying EBM when homogenous heat treatment and multiple reverse transformation treatments are conducted.

## 2. Experimental Procedure

### 2.1. Raw Materials and Metal-Additive-Manufactured Materials

The raw material was a gas-atomized powder (Sanyo Special Steel Co., Ltd, Hyogo, Japan) with a nominal composition of Co–28Cr–6Mo–0.11N (mass%); its particle size ranged from 45 to 150 μm, and D50 was ~60 μm. Rod materials were prepared using an EBM device (Arcam A2X, Arcam AB, Mölndal, Sweden). Sixteen rods were simultaneously fabricated on an SUS304 steel base plate (length of 150 mm, width of 150 mm, and thickness of 10 mm) arranged in four rows and four columns at 20 mm intervals, holding the build direction (z-axis) parallel to the longitudinal axis of rods. The electron-beam scan direction was parallel to the x- and/or y-directions. Each rod was 160 mm in height and 7 mm in diameter. According to Aoyagi et al. [18], we determined an optimum parameter set for fabrication. The preheating temperature was 1133 K, electron beam acceleration voltage 60 kV, beam current 3–18 mA, beam scanning speed 95–919 mm/s, line offset 260 μm, and layer thickness 70 μm. Table 1 lists the chemical composition of the EBM-fabricated materials.

### 2.2. Microstructure Evaluation and Phase Identification

The EBM-fabricated materials were cut using an electric discharge machine (HS-300, Brother Industries Ltd., Nagoya, Japan) to ensure that they were perpendicular to the building direction. The outer surface was observed using optical microscopy (VR-3200, Keyence, Osaka, Japan). The microstructure was analyzed by performing electron backscatter diffraction (EBSD, FEI XL30S-FEG, FEI Company, Hillsboro, OR, USA) at an acceleration voltage of 20 kV, and data analysis was conducted using an orientation image microscope system (TexSEM Laboratories Inc., Provo, UT, USA). Further microstructural observations were performed using transmission electron microscopy (TEM, JEM-2000EXII, JEOL, Akishima shi, Japan) at an acceleration voltage of 200 kV. X-ray diffraction (XRD, X’Pert MPD, Malvern Panalytical Ltd., Almelo, The Netherlands) using Cu Kα radiation was employed for phase identification.

### 2.3. Heat Treatment

**Figure 1:**
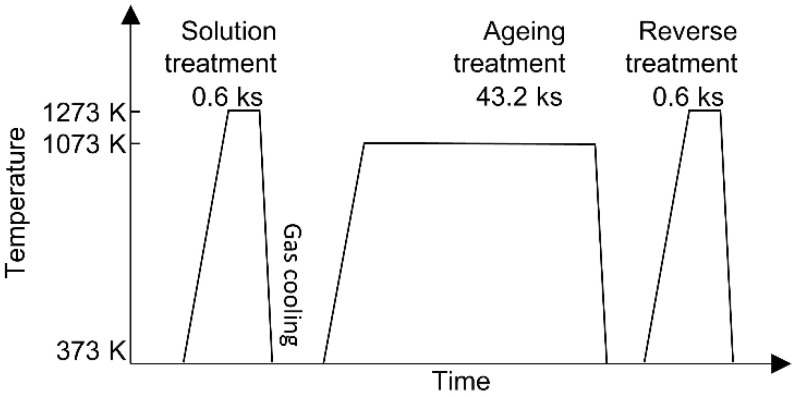
Schematic of heat treatment for EBM-fabricated materials.

**Figure 2:**
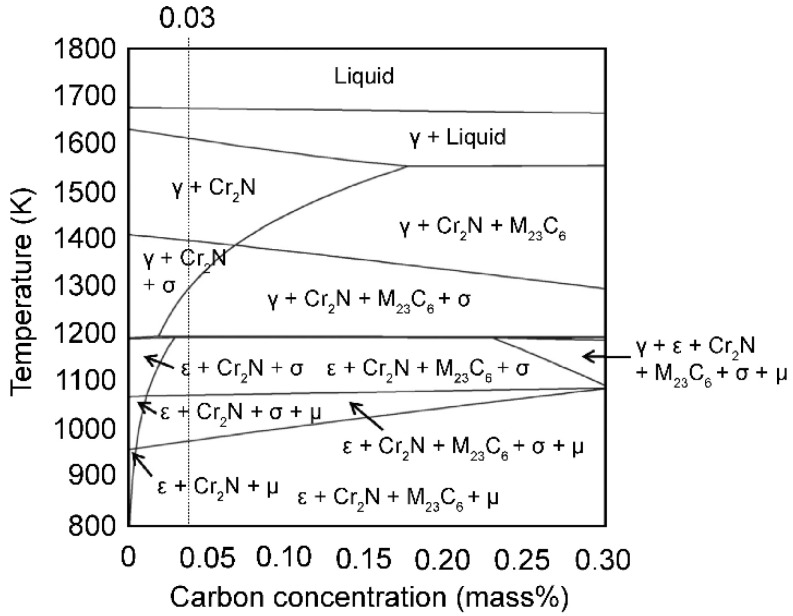
Phase diagram of Co–29Cr–6Mo–0.2N–xC (mass%) alloy. Adopted with permission from [19].

A heat-treatment furnace (Toei Scientific Industries Co., Ltd., Sendai, Japan), which was heated under an Ar gas atmosphere and cooled with Ar gas flow, was used to conduct repeated heat treatments, as shown in the schematic in Figure 1. For solution treatment (ST), the as-built (AsB) material was heated to 1273 K for 0.6 ks under an Ar atmosphere and then cooled with additional Ar gas. The ST material was then heated to 1073 K for 43.2 ks to perform aging treatment (AT). Thereafter, the AT material was heated to 1273 K for 0.6 ks to perform reverse transformation treatment (RT). The heat treatment from AT to RT was set as a single cycle, and three heat treatment cycles (3CT) were conducted. The condition was based on the calculated phase diagram of the Co–Cr–Mo alloy adopted from Yamanaka et al. [19], as shown in Figure 2.

### 2.4. Tensile Test

**Figure 3:**
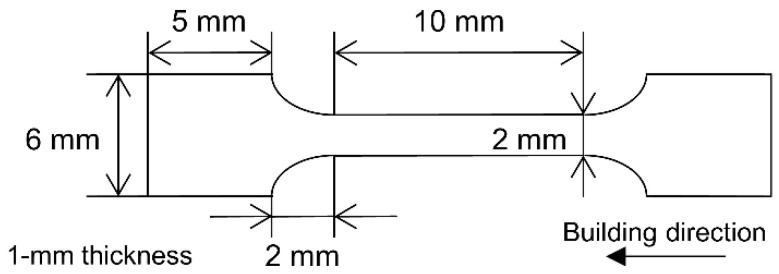
Schematic of the tensile test piece.

The EBM-fabricated rod was first cut into 1 mm-thick plates parallel to the longitudinal axis of the rod using a wire-cut electric discharge machine and then cut into a dog-bone shape having a gauge length of 10 mm, width of 2 mm, and thickness of 1 mm, as shown in Figure 3; next, surface polishing was performed. The conventional room-temperature tensile test was conducted using a universal testing machine (AUTOGRAPH DSS-10T, Shimadzu, Kyoto, Japan) at a strain rate of 1.7 × 10^−3^ s^−1^.

## 3. Results

### 3.1. Surface Morphology Observation of the AsB Material

**Figure 4:**
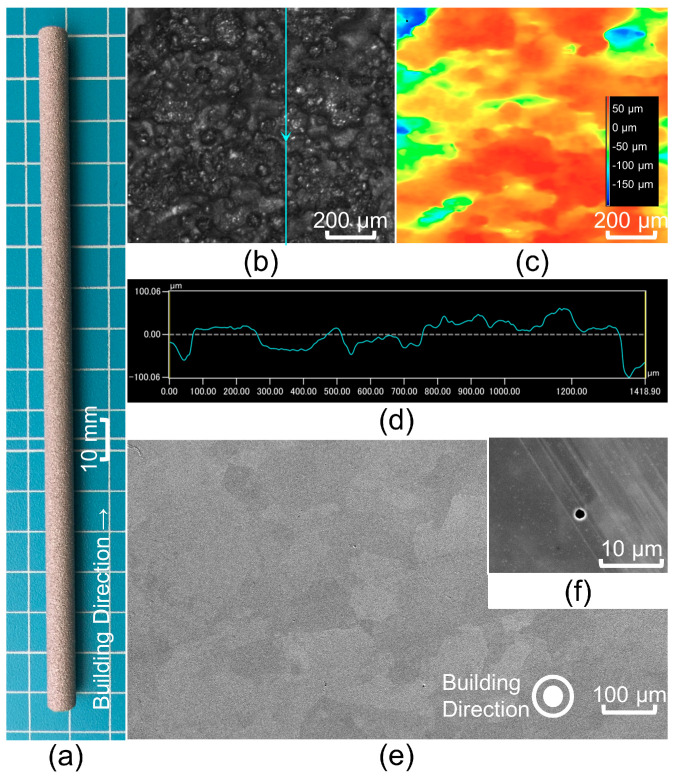
Optical and SEM observations of the AsB material. Optical image of the outer surface in low magnification (**a**) and high magnification (**b**). Height view (**c**) and line roughness profile (**d**) are indicated by the blue line in (**b**). SEM image for cross-section (**e**) with pore (**f**).

Figure 4 shows the optical and scanning electron microscopy (SEM) images of the AsB material. Figure 4a,b show low- and high-magnification optical images of the outer surface, respectively. Figure 4c shows the height profile of Figure 4b, and Figure 4d shows the surface roughness (Ra) of the line profile indicated by the blue line in Figure 4b. The adhesion of unmelted powder on the outer surface was confirmed because the molten pool formed using electron beam irradiation can trap adjacent powder particles [20]. The calculated Ra was 22.76 μm. The SEM image demonstrates the cross-section of the AsB material on the plane perpendicular to the fabrication direction. No major defects, such as non-melted zones or cracks, were observed. However, a few micro-meter-size pores were confirmed, as shown in Figure 4f. The pores were spherical and considered to be gas pores derived from the raw-material gas-atomized powder [21].

### 3.2. Phase Identification by Using XRD

**Figure 5:**
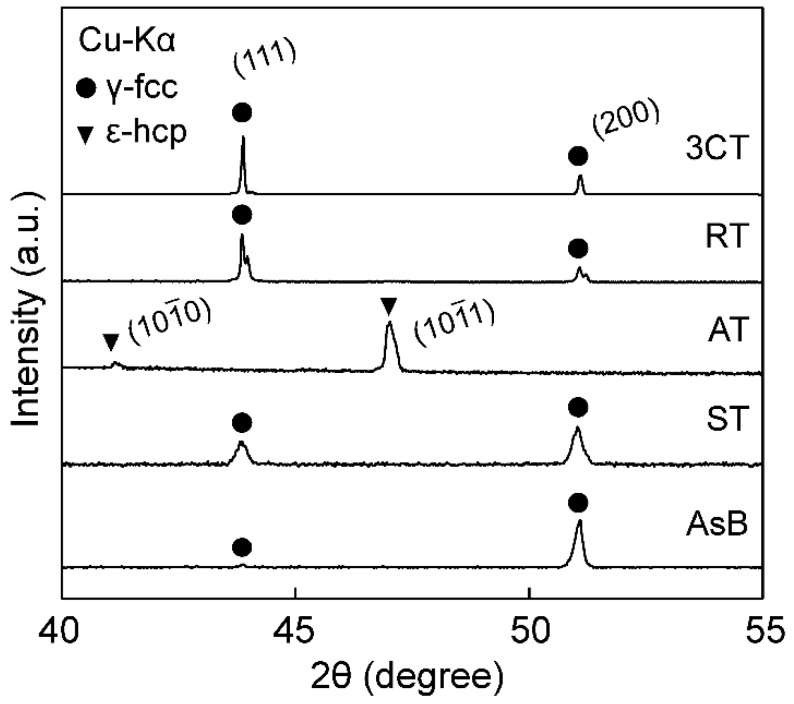
XRD patterns for a longitudinal cross-section of the EBM-fabricated materials: AsB, ST, AT, RT, and 3CT. ● for the γ-fcc phase and ▼ for the ε-hcp phase.

Figure 5 shows the XRD patterns of the AsB, ST, AT, RT, and 3CT materials. The XRD patterns for the AsB, ST, RT, and 3CT materials show peaks at (200) and (111) as the γ-FCC single phase. Conversely, the XRD pattern for the AT material shows peaks at $\left(10\bar{1}0\right)$ and $\left(10\bar{1}1\right)$ as the ε-HCP phase. This indicates that cooling with Ar gas maintained the high-temperature phases at 300 K, such as the high-temperature γ-FCC and low-temperature ε-HCP phases.

### 3.3. EBSD Analysis

**Figure 6:**
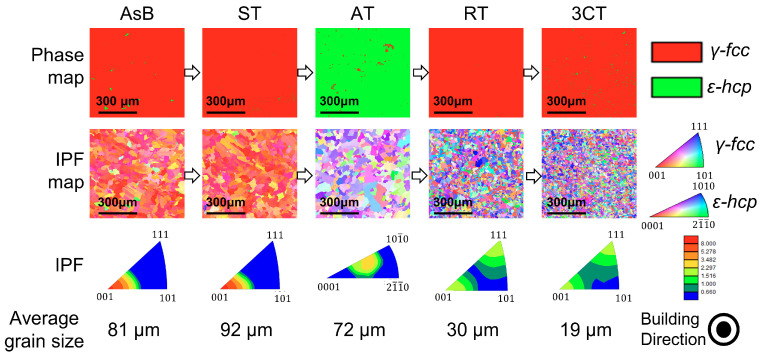
EBSD observations on the plane perpendicular to the fabrication direction after each heat treatment for EBM-fabricated materials: AsB, ST, AT, RT, and 3CT.

Figure 6 shows the phase map, inverse pole figure (IPF) map, IPF, and average grain size for the EBSD analysis of the materials before (AsB) and after each heat treatment (ST, AT, RT, and 3CT) on the plane perpendicular to the fabrication direction. The AsB material exhibits a γ-phase with a slight ε-phase in the phase map. It exhibits strong crystal anisotropy in which columnar grains grow along the building direction, as presented in the IPF map and IPF. The average grain size is approximately 80 µm. During the EBM process, the rapid melting and solidification of the metal as the electron beam scans and fuses the powder can result in the formation of columnar crystalline structures. These structures are characterized by elongated grains or crystals that grow perpendicular to the building surface. Columnar grains are typically observed in the direction of heat flow, and they can impact the mechanical properties of the final part. However, it is known that the mechanical properties of columnar grains are strong in the direction parallel to the elongation but weak in the direction perpendicular to the elongation. Therefore, columnar crystals do not have the same mechanical properties in all directions, which can be a drawback for common biological components.

The ST material exhibits the γ-phase with a slight ε-phase, and although no significant change in the crystal anisotropy is observed, the average grain size coarsens to >90 µm. The main purpose of ST is to dissolve and homogeneously disperse specific elements or phases within a material to achieve specific desired properties [22]. Accordingly, the AT at 900 to 1200 K allows complete transformation from γ- to ε-phase [23,24,25]. The AT material recrystallizes into an equiaxed low-temperature, stable single ε-phase with an average grain size of approximately 72 µm. However, although a slightly strong peak is observed in a specific direction in the IPF, no clear correlation exists between the (0001) plane of the ε-HCP phase and the (111) plane of the γ-FCC phase of the AsB and ST materials. Then RT allows transformation back from ε- to γ-phase with grain refinement effect at above 1200 K. The RT material recrystallizes into an equiaxed single γ-phase via the reverse transformation treatment, and the average grain size is reduced to approximately 30 µm. Furthermore, the 3CT material exhibits a grain refinement of approximately 20 μm because of repeated RT.

For the ε-phase slightly generated in the matrix γ-phase of the AsB material, the powder material is instantaneously melted during layer-by-layer melting and solidification such that the material temperature is above the liquidus temperature, and the actual material temperature can be expected to considerably exceed the set baseplate temperature of 1133 K. Moreover, the temperature after solidification should remain above 1133 K. Because the boundary temperature for the γ- and ε-phase is approximately 1173 K as per the phase diagram of the Co–29Cr–6Mo–0.2N–xC (mass%) alloy adopted from Yamanaka et al. (Figure 2) and the material temperature during fabrication changes from the liquidus temperature to near the boundary temperature, the γ → ε or ε → γ phase transformation is roughly considered to have occurred indeterminately during the fabrication process. However, as the γ-phase is dominant and the ε-phase is slightly generated, the material temperature during fabrication is expected to be higher than 1173 K.

### 3.4. Microstructure Observation

**Figure 7:**
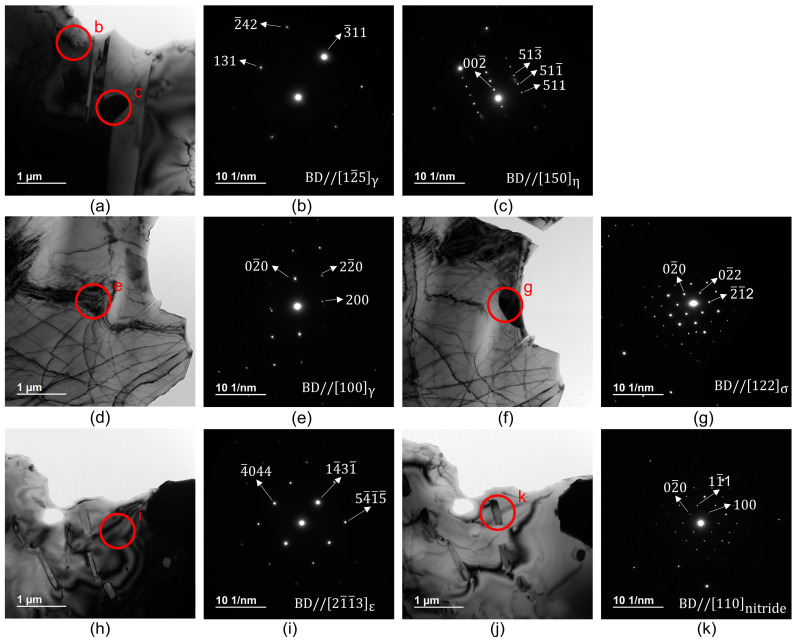
TEM image (**a**,**d**,**f**,**h**,**j**) and corresponding SAD patterns (**b**,**c**,**e**,**g**,**i**,**k**). A single γ-phase 3CT material is indicated in (**a**–**g**), and a single ε-phase AT material is indicated in (**h**–**k**).

Figure 7 shows the TEM images (a, d, f, h, j) and corresponding selected area diffraction (SAD) patterns (b, c, e, g, i, k) of the single γ- and ε-phase structures; the γ-phase structure is displayed in Figure 7a–g, and the ε-phase structure is depicted in Figure 7h–k. Many spherical precipitates, such as “c” in Figure 7a and “g” in Figure 7f, can be observed at the grain boundaries and in the γ-phase. Furthermore, electron diffraction patterns close to the η-phase (M_6_X-M_12_X, $Fd\bar{3}m$) and σ-phase (CoCr, $P4_{2/mnm}$) are shown in Figure 7e and Figure 7g, respectively. Many elongated precipitates, such as “k” in Figure 7j, can be observed in the grain boundaries and in the ε-phase, and their electron diffraction patterns close to the Cr2(C, N) phase (Cr_2_C_0.39_N_0.61_, Pbcn) are shown in Figure 7k. Although precipitates were expected to appear in the XRD patterns in Figure 5, the absence of the corresponding peaks may be ascribed to their low amount or magnitude being below the detection limit of the instrument.

Additionally, the phase transformation behavior of the Co–Cr–Mo alloy significantly changes after nitrogen is added, and this further suppresses the formation of the ε-phase and precipitation of the σ-phase owing to athermal martensitic transformation [25]. Kurosu et al. [8] reported that the γ → ε phase transformation at 800–1073 K produces a lamellar structure that consists of the ε-phase and chromium nitride formed from the metastable γ-phase and results in the precipitation of the molybdenum-enriched phase at the prior γ-grain boundary. Therefore, the grain growth of the ε-phase may be suppressed, and many precipitates formed immediately after the fabrication may remain, resulting in finer ε-phase grains. The result that the grain size is refined from 92 to 19 μm is roughly consistent with that of the reverse-transformation-treated Co–27Cr–5Mo–0.16N cast alloy reported by Kurosu et al. [8]. A majority of the precipitates of the fine-grain structure of the single γ-phase are the σ- and η-phases that precipitate and remain during fabrication. Furthermore, the surface integral rates of these two types of precipitates are reduced because of the RT. Thus, carbon, nitrogen, and silicon in the precipitates are solid-solved in the matrix because of long-term heat treatment. Simultaneously, the grain-growth-suppressing effect of the σ- and η-phases almost disappears, resulting in a stable grain size; this is also consistent with the reported eutectoid transformation from the γ-phase to the ε- and Cr_2_N phases in the N-containing Co–Cr–Mo alloy. Thus, the grain growth is suppressed because of the grain-boundary-pinning effect of the Cr_2_N phase during the reverse transformation to the γ-phase.

### 3.5. Mechanical Properties

**Figure 8:**
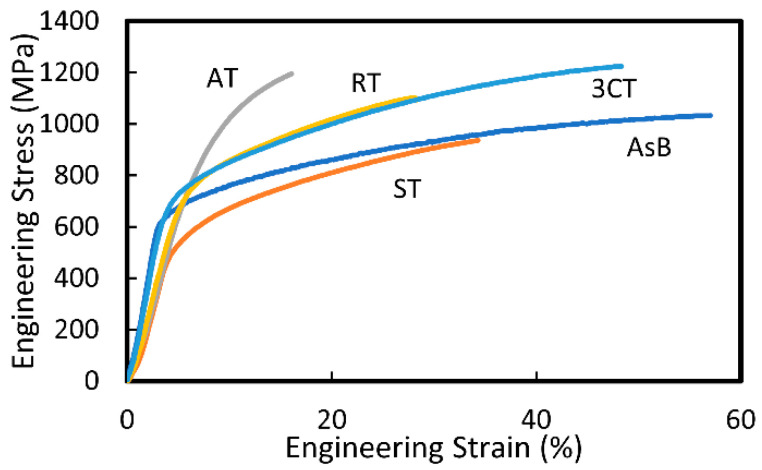
Tensile stress–strain curves for EBM-fabricated materials: AsB, ST, AT, RT, and 3CT.

Figure 8 shows the engineering tensile stress–strain curves of the AsB, ST, AT, RT, and 3CT materials, and the mechanical properties of these materials are summarized in Table 2. The yield strength, tensile strength, and elongation of the AsB material were 607 MPa, 1030 MPa, and 57%, respectively. As these values significantly exceed the yield strength (560 MPa), tensile strength (960 MPa), and elongation (20%) in the ASTM F75 standards, the AsB material can be treated as a standard material. The excellent strength and elongation can be attributed to the single γ-phase grains oriented in the <100> direction. This resulted in high apparent yield stress owing to the small Schmidt factor, which corresponded to the main slip system on $\left\{111\right\}<11\bar{0}>$ during the tensile test parallel to the fabrication direction. The yield strength, tensile strength, and elongation of the ST material were 472 MPa, 939 MPa, and 35%, respectively. Similar to the AsB material, the ST material had a columnar single γ-phase, which is a characteristic of EBM. However, the inferior mechanical properties were attributed to the grain coarsening (AsB: 81 μm → ST: 92 μm). The Bailey–Hirsch relationship indicates a proportional connection between the yield stress and dislocation density, which promotes the introduction of dislocations as the grains become smaller; conversely, the dislocation density decreases as the grains become larger [26,27,28]. As a result, the yield stress decreases, and dislocation strengthening decreases with grain coarsening. The yield strength, tensile strength, and elongation of the AT material were 740 MPa, 1195 MPa, and 16%, respectively. As the AT material had a single ε-phase during the γ → ε phase transformation, unlike the AsB and ST materials with a single γ-phase, its mechanical behavior was strongly influenced by the improved tensile and yield strengths with worsened ductility. The yield strength, tensile strength, and elongation of the RT material were 615 MPa, 1089 MPa, and 28%, respectively, whereas those of the 3CT material were 655 MPa, 1234 MPa, and 45%, respectively. Unlike the AsB and ST materials, which had columnar γ-phases, the RT and 3CT materials exhibited equiaxed γ-phases; thus, isotropic mechanical properties can be achieved. In particular, the 3CT material had finer grains than the RT material, and its mechanical properties were superior because the mechanical properties of Co–Cr–Mo alloys are reported to be significantly improved by refining the grain size using hot forging [8,9,10]. We considered grain refinement as one of the strengthening mechanisms that can improve the tensile properties of materials, where the Hall–Petch law was applied to the grain size and yield stress [27,28,29]. Hence, the improved mechanical properties of the 3CT material can be attributed to grain refinement.

**Figure 9:**
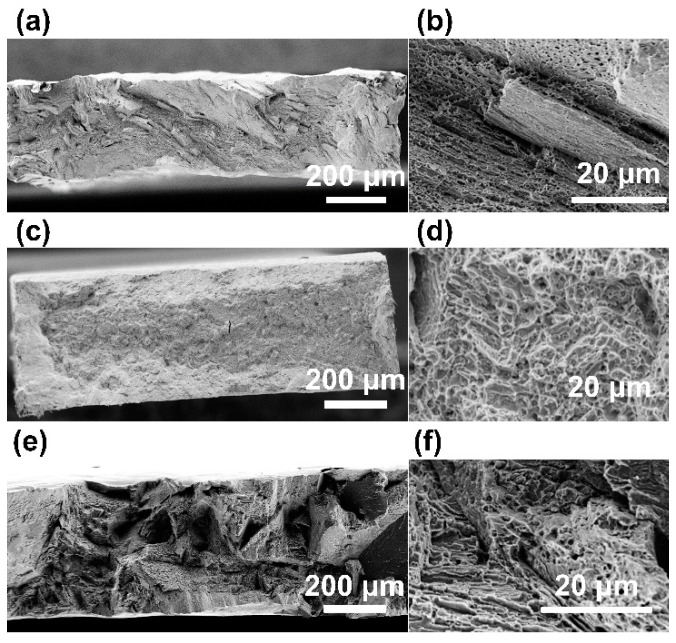
Surface morphology of AsB (**a**,**b**), 3CT (**c**,**d**), and AT (**e**,**f**) materials after tensile tests. (**a**,**c**,**e**) and (**b**,**d**,**f**), respectively, represent low- and high-magnification images.

Figure 9 shows the fracture surfaces of the AsB (Figure 9a,b), 3CT (Figure 9c,d), and AT (Figure 9e,f) materials after tensile tests, and Figure 9a,c,e and Figure 9b,d,f present the low- and high-magnification images, respectively. Figure 9a displays a fibrous fracture surface with fine irregularities perpendicular to the tensile direction, indicating the predominant ductility contribution of the γ-phase. However, Figure 9b demonstrates steps owing to cleavage fractures with a specific orientation, indicating the brittleness contribution of the slight ε-phase. Therefore, the ductility contribution of the γ-phase is dominant during the tensile fracture of AsB materials, whereas the ε-phase contributes to brittleness. Figure 9c shows a fibrous fracture surface with fine irregularities perpendicular to the tensile direction. Moreover, only a dimple fracture, indicating ductile fracture, can be observed in Figure 9d, which shows the predominant ductile contribution of the γ-phase. Figure 9e shows an intergranular fracture surface due to the ε-phase.

## 4. Discussion

### 4.1. Mechanism of the RT

Generally, the γ-phase of the FCC structure is stable in the high-temperature region (approximately 1173 K and higher), and the ε-phase of the HCP structure is stable in the low-temperature region (approximately 1173 K and lower). In addition, the σ-phase is formed when chromium, molybdenum, and nitrogen are added, whereas a martensite ε-phase is formed through athermal martensitic transformation when the material is rapidly cooled from the γ-phase stable region. The martensite ε-phase is formed through a strain-induced martensitic transformation due to tension and compression.

Sun et al. [30] reported a directional relationship between the {111} plane of the γ-phase and the (0001) plane of the ε-phase in EBM-fabricated Co–Cr–Mo–C–N alloys. These results suggested an isothermal martensitic transformation from the γ-phase to the ε-phase, in which the orientation relationship of Shoji–Nishiyama $\left((111)\gamma //(0001)\varepsilon ,[\bar{1}10]\gamma //[11\bar{2}0]\varepsilon \right)$ was maintained. Additionally, Kurosu et al. reported a massive transformation dominant during the γ → ε phase transformation at 973–1173 K for the Co–29Cr–6Mo alloy [8]. After the precipitation of the σ-phase at the grain boundary of the prior γ-phase due to AT, the ε-phase grain is formed from the prior grain boundary because of the massive transformation in the two adjacent grains. Thereafter, Kurosu et al. indicated that the γ → ε phase transformation at 1073 K competed with the massive and isothermal martensitic transformations in the Co–29Cr–6Mo alloy [8]. That is, the grain boundary migration of new grains was halted because of the collision between the massive grains, and the σ-phase was formed at the interface between the new grains and at the interface between the matrix and new grains. Furthermore, a minute striation of the ε-phase due to the isothermal martensitic transformation that satisfies the aforementioned Shoji–Nishiyama relationship was confirmed in the region of 700–1073 K.

In the ε-phase structure of the fabricated Co–Cr–Mo–N alloy, although the orientation was biased in this study, the ${\left(111\right)}_{\gamma }$ and ${\left(0001\right)}_{\varepsilon }$ planes and the ${\left[\bar{1}10\right]}_{\gamma }$ and ${\left[11\bar{2}0\right]}_{\varepsilon }$ directions did not exhibit a clear orientation relationship. This may be because the applied preheating temperature of 1133 K is close to the massive transformation-initiation temperature. Therefore, the ε-phase structure induced by preheating contained a negligible number of grains that were derived from isothermal martensitic transformation, and most grains were randomly oriented because of the diffusion transformation. Therefore, the ε-phase grains were formed at multiple nucleation sites, including those in the grain boundaries of the prior γ-phase and dendrite interface before the ε-phase transformation.

Another perception is that the phase transformation behavior of the Co–Cr–Mo alloy significantly changes after nitrogen is added, thus stabilizing the γ-phase and suppressing the formation of the ε-phase and precipitation of the σ-phase owing to athermal martensitic transformation [25]. Kurosu et al. [8] reported that the γ → ε phase transformation at 800–1073 K produces a lamellar structure that consists of the ε-phase and chromium nitride resulting from the metastable γ-phase and causes the precipitation of the molybdenum-enriched phase at the prior γ-grain boundary. The time-temperature-transformation (TTT) curve diagram [19] of the Co–27Cr–5.5Mo–0.16N alloy prepared by Kurosu et al. demonstrated that the constituent phase should be a single γ-phase at a preheating temperature of 1133 K. However, our results are in agreement with the phase distribution of the EBM-fabricated Co–28Cr–6Mo–0.23C–0.2N alloy reported by Sun et al. [31]. This may be because of the Co–Cr–Mo–N alloy composition used in this study. Kurosu et al. reported that in the TTT curve diagrams of the Co–Cr–Mo and Co–Cr–Mo–N alloys, the earliest temperatures at which the γ → ε phase transformation started were 1173 K and 1073 K, and the corresponding transformation-initiation times were 1.8 ks and 216 ks, respectively [32]. This can be attributed to the reduced free energy of the γ-phase because the addition of nitrogen is considered to lower the free energy, $G^{\gamma }$, and reduce the free energy difference between $G^{\gamma }$ and $G^{\varepsilon }$. In other words, the incubation period of the ε-phase is considered to become longer as $∆Gv=\left|G^{\varepsilon }-G^{\gamma }\right|$ becomes smaller. The incubation period [30,33,34] is the time until precipitation or transformation starts, that is, the time until nucleation through precipitation or transformation starts. The incubation period, τδ, is expressed as $T_{\delta }=(8kT\sigma a^{4})/(v_{a}^{2}{\left(\Delta G_{v}\right)}^{2}Dx_{B})$, where k is the Boltzmann constant, T is temperature, σ is the interfacial energy per unit volume, a is the lattice constant, $v_{a}$ is the volume per atom in the cluster, D is the diffusion coefficient, and $\Delta G_{v}$ is the free energy difference per unit area in nucleation. Therefore, the incubation period of the ε-phase is prolonged with the addition of nitrogen.

Nevertheless, the incubation period of the ε-phase in this study may be shortened mainly because we added only 0.1 mass% nitrogen, whereas the reported Co–Cr–Mo–N alloy was 0.16 mass%. Furthermore, Kurosu et al. indicated that in the Co–29Cr–6Mo alloy, the γ → ε phase transformation at 1073 K competed with the massive and isothermal martensitic transformations [8]. However, in this study, no clear directional relationship existed between the (111) IPF of the γ-FCC phase and the (0001) plane of the ε-HCP phase, suggesting that massive transformation was dominant in the γ → ε phase transformation at 1073 K in the EBM-fabricated material. Therefore, ε-phase grains were formed because of the diffusion transformation at multiple nucleation sites, including those in the grain boundaries of the prior γ-phase and dendrite interface before the ε-phase transformation. Furthermore, the grain growth of the ε-phase may be suppressed, and many precipitates formed immediately after the fabrication may remain, resulting in finer ε-phase grains.

In this study, the grain size was refined from 92 to 19 μm after the three-time RT. This result is roughly consistent with that of the reverse-transformation-treated Co–27Cr–5Mo–0.16N cast alloy reported by Kurosu et al. [8]. Most of the precipitates of the fine-grain structure of the single γ-phase were the σ- and η-phases that precipitate and remain during fabrication. Furthermore, the surface integral rates of these two types of precipitates were reduced through multiple RTs; thus, carbon, nitrogen, and silicon in the precipitates were solid-solved in the matrix because of long-term heat treatment. Simultaneously, the grain-growth-suppressing effect of the σ- and η-phases almost disappeared, causing the grain size to stabilize; this is also consistent with the reported eutectoid transformation from the γ-phase to the ε- and Cr_2_N phases in the N-containing Co–Cr–Mo alloy. That is, the growth of the grains was suppressed because of the grain-boundary-pinning effect of the Cr_2_N phase during the reverse transformation to the γ-phase.

### 4.2. Enhancement of Mechanical Properties

Kurosu et al. reported that in Co–Cr–Mo alloys with nitrogen additions, γ-phase grain refinement is possible by applying heat treatment via the reverse transformation and eutectic transformation from the γ-phase to the ε- and Cr_2_N phases [8]. Grain refinement is one of the strengthening mechanisms for improving the tensile properties of materials, and the Hall–Petch law holds for grain size and yield stress [29,35,36,37]. Thus, the relationship between the yield strength and grain size conforms to the Hall–Petch law, except for single-phase AT materials. Even in Co–Cr–Mo alloys, significant improvement in the mechanical properties as a result of ultrafine grain size through hot forging has been reported [38,39].

**Figure 10:**
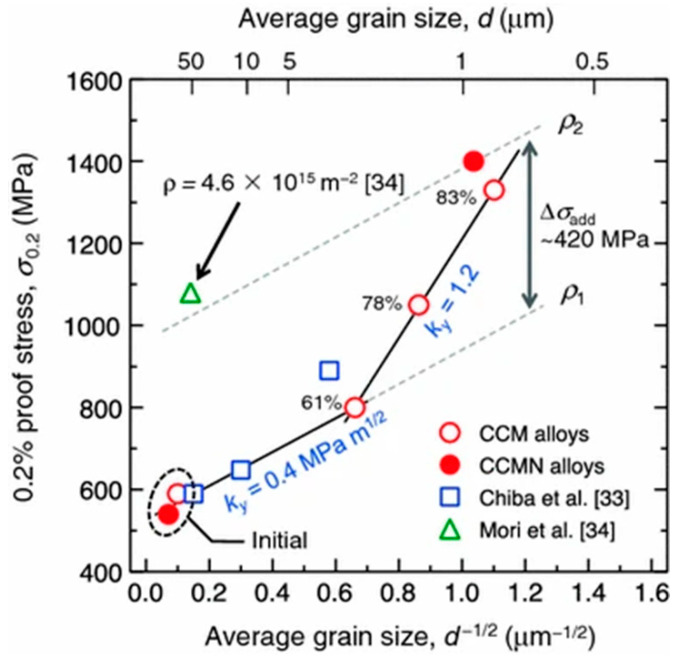
Yield (0.2% proof) stress of Co–Cr–Mo alloy as a function of average grain size. Reprinted from [40].

Figure 10 illustrates the relationship between the average grain size and yield stress in the heat-treated and hot-forged Co–Cr–Mo alloy materials that were prepared by Yamanaka et al. [40]. Because conducting heat treatment on EBM-fabricated objects having complex shapes, such as artificial joints, is difficult, a homogeneous material with excellent mechanical properties needs to be obtained using only heat treatment. The improvement in the mechanical properties observed in the present study is partly because of grain refinement resulting from the reverse transformation treatment.

## 5. Conclusions

This study focused on the biomedical Co–28Cr–6Mo–0.11N alloy fabricated by EBM. The phase transformation and precipitation behavior after multiple reverse transformations and the effects of these transformations on the mechanical properties of the alloy were investigated. The conclusions of this study are as follows:

1. The dominant phase transformation behavior was observed in the apparent γ → ε phase transformation at 1073 K. The added nitrogen either formed a nitride or was present in a solid solution. The incubation period of the ε-phase in this study may have been shortened because we added only 0.1 mass% nitrogen. Further, ε-phase grains were formed through diffusion transformation at multiple nucleation sites, including those in the grain boundaries of the prior γ-phase and dendrite interface before the ε-phase transformation.
2. Because no fracture originated from precipitates, such as the σ-, η-, and Cr_2_N phases, the impact of precipitates on tensile properties was considered to be small. Carbon, nitrogen, and silicon in the precipitate were solid-solved in the matrix because of long-term heat treatment. Simultaneously, the grain-growth-suppressing effect of the σ- and η-phases almost disappeared, causing the grain size to stabilize. Subsequently, the growth of the grains was suppressed because of the grain-boundary-pinning effect of the Cr_2_N phase during the reverse transformation to the γ-phase.
3. The grain size of the 3CT material was refined to 19 μm, which was approximately 1/4 times that of the AsB material. The yield strength, tensile strength, and elongation of the 3CT material were 655 MPa, 1234 MPa, and 45%, respectively. The performance was equivalent to that of the cast material subjected to the same treatment but significantly exceeded the ASTM F75 standards (560 MPa, 960 MPa, and 20%).
4. Furthermore, the yield strength of the single ε-phase was 740 MPa, and that of the single γ-phase after three reverse transformations was 655 MPa, suggesting that a higher yield strength could be obtained using a mixed phase of the ε-phase and γ-phase structure than using the single γ-phase structure.

## Figures and Tables

**Table 1:** Chemical composition of the EBM-fabricated Co–28Cr–6Mo–0.11N alloy built-in mass percent.

Cr	Mo	Fe	Si	Mn	C	O	N	Co
27.5	5.5	0.18	0.31	0.5	0.03	0.01	0.1	Bal.

**Table 2:** Mechanical properties of EBM-fabricated specimens: AsB, ST, AT, RT, and 3CT. ASTM F75 requirements are presented for comparison.

	AsB	ST	AT	RT	3CT	ASTM F75
Yield strength (MPa)	607 ± 6	472 ± 21	740 ± 10	615 ± 25	655 ± 32	≥560
Tensile strength (MPa)	1030 ± 3	939 ± 10	1195 ± 8	1089 ± 20	1234 ± 82	≥960
Elongation (%)	57 ± 0	35 ± 5	16 ± 2	28 ± 3	45 ± 9	≥20

## Data Availability

Not applicable.
